# *Culex tarsalis* Vitellogenin Gene Promoters Investigated *In Silico* and *In Vivo* Using Transgenic *Drosophila melanogaster*

**DOI:** 10.1371/journal.pone.0088994

**Published:** 2014-02-25

**Authors:** Song Chen, Jason L. Rasgon

**Affiliations:** 1 Department of Entomology, The Center for Infectious Disease Dynamics and the Huck Institutes of The Life Sciences, Millennium Science Complex, The Pennsylvania State University, University Park, Pennsylvania, United States of America; 2 The W. Harry Feinstone Department of Molecular Microbiology and Immunology, Bloomberg School of Public Health, Johns Hopkins University, Baltimore, Maryland, United States of America; New Mexico State University, United States of America

## Abstract

**Introduction:**

Genetic modification, or transgenesis, is a powerful technique to investigate the molecular interactions between vector-borne pathogens and their arthropod hosts, as well as a potential novel approach for vector-borne disease control. Transgenesis requires the use of specific regulatory regions, or promoters, to drive expression of genes of interest in desired target tissues. In mosquitoes, the vast majority of described promoters are from *Anopheles* and *Aedes* mosquitoes.

**Results:**

*Culex tarsalis* is one of the most important vectors of arboviruses (including West Nile virus) in North America, yet it has not been the subject of molecular genetic study. In order to facilitate molecular genetic work in this important vector species, we isolated four fat body-specific promoter sequences located upstream of the *Cx. tarsalis* vitellogenin genes (*Vg1a*, *Vg1b*, *Vg2a* and *Vg2b*). Sequences were analyzed *in silico* to identify requisite *cis*-acting elements. The ability for promoter sequences to drive expression of green fluorescent protein (*GFP*) *in vivo* was investigated using transgenic *Drosophila melanogaster*. All four promoters were able to drive *GFP* expression but there was dramatic variation between promoters and between individual *Drosophila* lines, indicating significant position effects. The highest expression was observed in line *Vg2b*L3, which was >300-fold higher than the lowest line *Vg1a*L2.

**Conclusions:**

These new promoters will be useful for driving expression of genes of interest in transgenic *Cx. tarsalis* and perhaps other insects.

## Introduction

Some genetic modification-mediated approaches for control of mosquito-borne diseases are based on deploying transgenic mosquitoes expressing genes that will affect mosquito vectorial capacity [1]. An essential step in this process is to identify suitable stage- and tissue-specific promoters to drive expression of anti-pathogen genes of interest, work on which has been primarily focused on midgut, salivary gland and fatbody promoters of *Anopheles* and *Aedes* mosquitoes. The promoter regions of the *Anopheles gambiae* carboxypeptidase (***AgCP***) and *Aedes aegypti* carboxypeptidase (*AeCP*) genes have been isolated and shown to drive midgut-specific bloodmeal-inducible gene expression [2]. The regulatory regions of the ***Ae. aegypti*** salivary gland specific maltase-like I (***Mal***I) and apyrase (***Apy***) genes were shown to direct tissue-specific expression of a luciferase reporter gene in transgenic ***Ae. aegypti*** [3]. The promoter fragments of *An. gambiae* female salivary gland-specific genes *AgApy* and *D7r4* directed expression of a *LacZ* reporter gene in the *An. stephensi* salivary gland [4]. A female salivary gland-specific promoter for a gene encoding anopheline antiplatelet protein (*AAPP*) isolated from *An. stephensi* was able to drive expression of DsRed in the salivary glands [5]. Several fatbody specific promoters have also been reported. The ***Ae. aegypti*** vitellogenin gene promoter was successfully used for fatbody expression and secretion of defensin into the hemolymph [1], [6]. A 1.7 kb promoter fragment of *An. gambiae* vitellogenin gene (*VgT2*) was able to direct expression of green fluorescent protein (*GFP*) in a tissue-, stage-, and sex-specific manner in transgenic *An. stephensi* [7].

By using transgenic *Drosophila*, some regulatory regions isolated from non-*Drosophila* insects (ranging from flies to lepidopterans) have been confirmed to be functional in *Drosophila*, providing evidence for transcriptional regulation similarities between *Drosophila* and non-*Drosophila* insects. The regulatory elements in promoter regions of the midgut-specific carboxypeptidase gene in *Simulium* (blackflies), and trypsin genes in *An. gambiae* were recognized and able to drive expression of reporter genes in a gut-specific manner in *Drosophila* [8]–[9]. The promoter regions of the apyrase and D7-related genes expressed in *An. gambiae* salivary glands were able to drive tissue-specific reporter gene expression in transgenic *Drosophila* [4], [10]. A regulatory region of the fatbody specific vitellogenin gene could direct the expression of the reporter in a correct stage- and tissue-specific manner in *Drosophila* similar to the endogenous *Drosophila* yolk protein genes [6]. A portion of the 5′-flanking region of the female-specific hexamerin gene, ***Hex-1.2***, from the mosquito ***Ochlerotatus atropalpus*** drove expression of the ***luciferase*** reporter gene in the ***D. melanogaster*** fatbody [11]. Even in more divergent lepidopterans, some gene regulatory regions are also recognized by *Drosophila* [12]–[13]. Therefore, *Drosophila* is a useful system for characterizing the transcriptional regulation of genes from non-*Drosophila* insects. Moreover, *Drosophila* transformation is simpler than mosquito transformation and is especially useful to study regulatory regions from insect species for which transformation has not been achieved.

*Culex tarsalis* is one of the most important vectors of arboviruses in North America. It has been associated with or is capable of transmission of West Nile Virus [14]–[18], Western Equine Encephalitis virus [19]–[21], St. Louis Encephalitis virus [20]–[21], Japanese Encephalitis virus, Venezuelan Equine Encephalitis virus [22] and Rift valley fever virus [23]. To our knowledge, no tissue-specific promoters have been isolated or tested from this significant vector species. Previously we isolated four vitellogenin genes (*Vg1a*, *Vg1b*, *Vg2a* and *Vg2b*) from *Cx. tarsalis* [24]–[25]. In this study we cloned, bioinformatically and functionally characterized the four vitellogenin gene promoters from *Cx. tarsalis*. These putative promoter sequences were used to drive a reporter gene (*GFP*) in the fat bodies of transgenic *D. melanogaster*. All 4 sequences were functional in *Drosophila*. There was dramatic variation between promoters and between individual *Drosophila* lines, indicating significant position effects. These promoter sequences will be useful for future molecular genetic studies in this important mosquito species.

## Results

### Cloning and *in silico* analysis of the 5′ putative regulatory regions of four vitellogenin genes from *Cx. Tarsalis*

The transcription factor binding sites (GATA, C/EBP, EcRE) which are required for tissue- and stage-specific vitellogenin expression in mosquito vitellogenin genes are usually located approximately 2 kb 5′ upstream of the initial vitellogenin transcriptional start site [1]. Four vitellogenin genes from *Cx. tarsalis* were isolated previously [24]–[25]. In this study, we cloned the putative 5′ regulatory regions for the four distinct vitellogenin genes using a genome walking approach [24]. Isolated sequences were 3159-bp, 1976-bp, 2950-bp and 2133-bp for *Vg1a*, *Vg1b*, *Vg2a* and *Vg2b*, respectively. Sequences were deposited into GenBank under accession numbers KF271781 - KF271784. Analysis showed that the sequences of the two *Vg1* promoters were highly conserved in the region from translation start codon “ATG” to 123-bp upstream, then increased in divergence. Likewise, sequences of the two *Vg2* promoters were highly-conserved in the region from “ATG” to 166-bp upstream, then increased in divergence. Sequences between the *Vg1* and *Vg2* regulatory regions were highly divergent.

**Figure 1:**
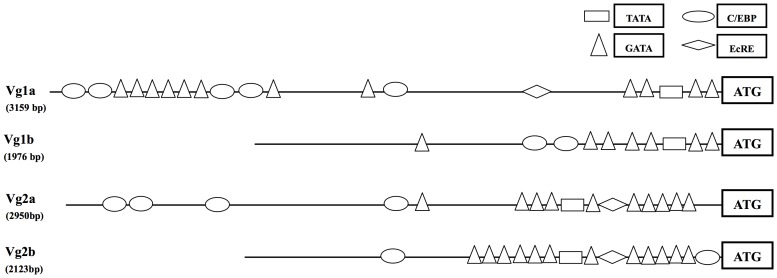
Schematic illustration of putative of regulatory elements in the promoter regions of the four *Culex tarsalis* vitellogenin genes.

Despite this divergence, rtPCR indicated that all four vitellogenin promoters had similar expression profiles and were active both pre- and post-bloodmeal [25], suggesting that they may share similar *cis*-regulatory elements. We analyzed these sequences to identify putative responsive elements. Even with highly divergence sequences, most of the transcription factor binding sites (GATA, TATA, C/EBP, EcRE), which are required for tissue- and stage- specific vitellogenin expression in the *Ae. aegypti Vg1* gene could be found in all four *Cx. tarsalis* vitellogenin promoters. Differences between each vitellogenin promoter were also identified (Figure 1).

### Activity analysis of four vitellogenin promoters in transgenic *D. melanogaster*

Germline transformation of *Cx. tarsalis* has not yet been achieved, so we used transgenic *D. melanogaster* to investigate the function of the *Cx*. *tarsalis* vitellogenin promoters *in vivo*. We designed 4 piggyBac constructs that each had the general organization: pBac[3XP3-DsRed-CxVg-promoter-EGFP] and generated transgenic *Drosophila* by embryonic microinjection with pBac helper plasmid. Three lines for each promoter were randomly selected for reporter gene expression analysis.

**Figure 2:**
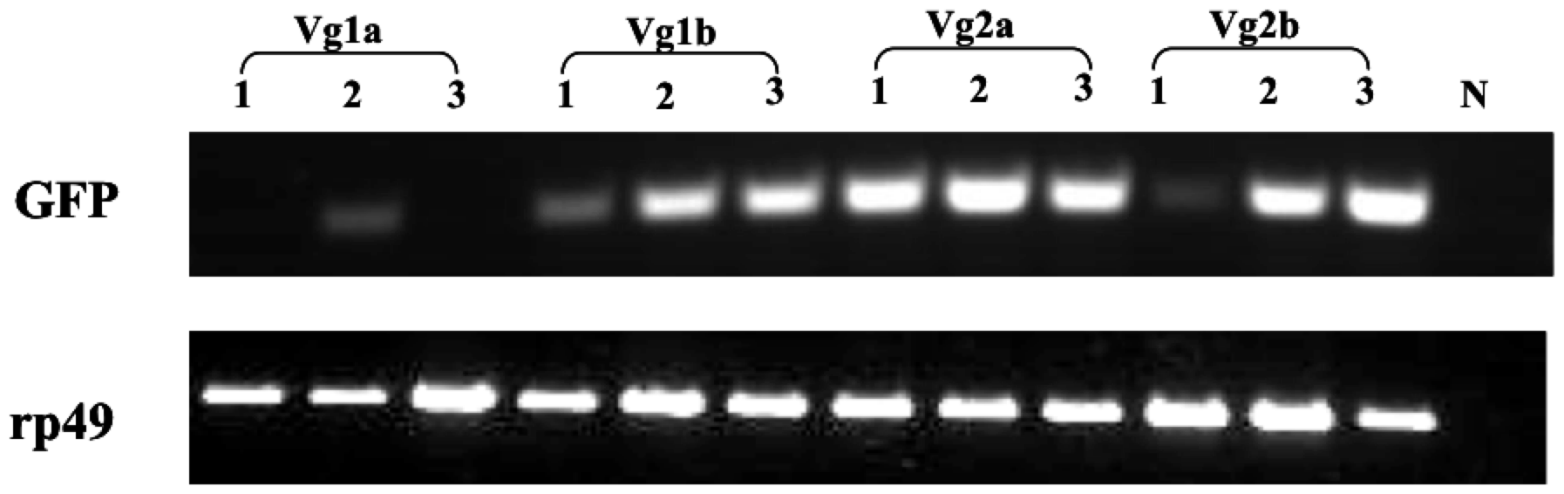
Expression of GFP gene in transgenic *Drosophila* by RT-PCR. Three independent lines for each promoter were assayed. The constitutive ribosomal protein 49 gene (*rp49*) in *D. melanogaster* was examined as an endogenous control. N  =  negative control.

**Figure 3:**
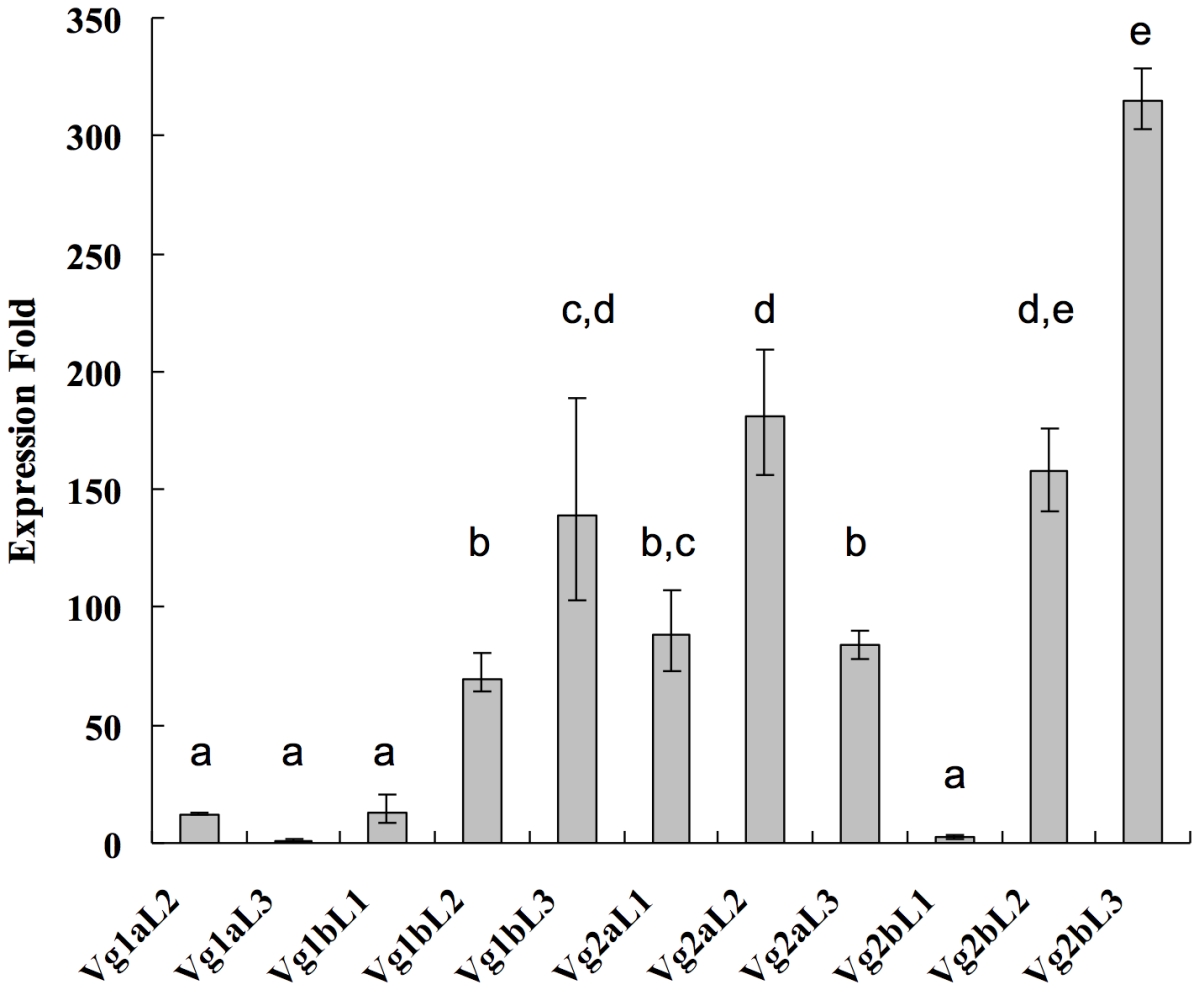
Variation in reporter gene (GFP) expression among transgenic *Drosophila melanogaster* lines. Relative expression levels of GFP were normalized to the expression of ribosomal protein 49 (***rp49***). Expression folds are calculated by comparison to line Vg1aL3, which had the lowest detectable GFP expression. Error bars represent standard errors. Letters represent statistical differences.

To monitor the expression level of *GFP* gene, RT-PCR and qRT-PCR analysis were employed. The results showed that *GFP* expression in *D. melanogaster* fat bodies was observed when driven by all *Cx. tarsalis* promoters, but expression levels were highly variable between different vitellogenin promoters and between different independent fly lines of the same promoter (Figures 2 and 3). In general, *GFP* expression driven by *Vg2a* and *Vg2b* promoters was higher than when driven by *Vg1a* and *Vg1b* promoters. The highest expression was observed in line *Vg2b*L3, which had 315-fold higher *GFP* expression compared to line *Vg1a*L3, the line with the lowest detectable *GFP* expression (no expression was detectable in line *Vg1a*L1). These results show that different lines transformed with the same promoter sequence had highly variable reporter gene expression, indicating position effects due to the integration site of the transposon in the *Drosophila* genome.

## Discussion

We isolated four putative promoter regions of vitellogenin genes from *Cx. tarsalis* and their function was analyzed using transgenic *D. melanogaster*. All four promoter sequences were able to drive reporter gene expression in *Drosophila* fat bodies, suggesting that these regulatory regions contain sufficient *cis*-acting elements for the correct expression of the gene. These analyses also demonstrated a conservation of regulatory element function between *D. melanogaster* and *Cx. tarsalis*, two distantly related species.

Our results suggest that position effects occur due to the site of integration of the transposon in the *Drosophila* genome. Position effects are very common and can result from novel enhancer/silencer-promoter interactions [26]–[30]. In *Drosophila*, positive position effects have been detected by inserting a transgene with a weak promoter near an enhancer element where high-level expression detected is due to the effect of the enhancer. In other instances, transcription is downregulated due to the negative action of neighboring silencing elements silencer.

The lowest activity was detected in *Drosophila* lines containing *Vg1a* promoter constructs, although this promoter contains the longest 5′ flanking fragment size (3.1 kb). It is possible that all three lines we used in the study had strong negative position effects due to (1) silencers near their insertion sites, although this regulatory region contains sufficient cis-elements for expression, (2) the *cis*-regulation elements controlling the expression of the vitellogenin gene may be located in 3′ regulatory region that is not included in the construct, or (3) the sequence we identified may contain repressor elements. Deletion analysis will help to clarify this in future studies.

To date, molecular genetic studies in *Cx. tarsalis* have been lacking compared to other mosquito species. These 4 identified promoter sequences will be useful for transgenic studies in this important vector insect.

## Materials and Methods

### Ethics statement

Mosquitoes were bloodfed on commercially obtained chicken blood using a membrane feeder.

### Mosquitoes

*Cx. tarsalis* used in this study were from the KNWR colony, which was collected in the Kern National Wildlife Refuge (KNWR) in Kern County, California [25]. Larvae were reared at a standard density of 200 larvae/pan and fed a 1∶2∶2 blend of fish food, rabbit pellets and bovine liver extract. Adults were maintained on 10% sucrose solution at 27±1°C and 80±5% relative humidity with a 14 h/10 h light/dark cycle.

### Genomic DNA extraction and partial coding sequence isolation

Genomic DNA was isolated from female adults using the procedure previously described [24]. Based on the available *An. gambiae*, *Ae. aegypti* and *Cx. pipiens* vitellogenin sequences from GenBank, primers were designed to PCR-amplify the partial coding sequence at the 5′ end of the genes (Table 1). PCR products were cloned using the TOPO TA cloning kit for sequencing (Invitrogen).

**Table 1:** PCR primers list.

Gene	Forward Primer(5′->3′)	Reverse Primer(5′->3′)	Amplicon Size (bp)
Vg1a promoter fragment	AGCTGGCCGGCCAGACAACTAAGACGTCCATG	GTTGATCGTTGAGGCAGGCAG	3159
Vg1b promoter fragment	CTGGCCGGCCATCCTCGTAACTCCTACCAC	GTTGATCGTTGAGGCAGGCAG	1976
Vg2a promoter fragment	AGCTGGCCGGCCGATGTGCAGCCATCCCCTTAG	CACGATCACTCGAGCTTTTTTCTTCAC	2950
Vg2b promoter fragment	AGCTGGCCGGCCGATGTTTTGCCTAAGACCTTTTG	CACGATCACTCGAGCTTTTTTCTTCAC	2133
Vg1ap+GFP+BGHR	AGCTGGCCGGCCAGACAACTAAGACGTCCATG	AGCTGGCCGGCC CCTAGAGCCCCAGCTGGTTC	4197
Vg1bp+GFP+BGHR	CTGGCCGGCCATCCTCGTAACTCCTACCAC	AGCTGGCCGGCC CCTAGAGCCCCAGCTGGTTC	3047
Vg2ap+GFP+BGHR	AGCTGGCCGGCCGATGTGCAGCCATCCCCTTAG	AGCTGGCCGGCC CCTAGAGCCCCAGCTGGTTC	4021
Vg2bp+GFP+BGHR	AGCTGGCCGGCCGATGTTTTGCCTAAGACCTTTTG	AGCTGGCCGGCC CCTAGAGCCCCAGCTGGTTC	3204
GFP (for RT-PCR)	AGGTGATGCTACATACGGAAAG	CATGCCATGTGTAATCCCAG	598
GFP (for qPCR)	TACAAGACGCGTGCTGAAGT	CAATGTTGTGGCGAATTTTG	199
rp49	ATCGGTTACGGATCGAACAA	GACAATCTCCTTGCGCTTCT	165

### Genomic DNA extraction, 5′ promoter region isolation and bioinformatics analysis of putative transcriptional factors binding site

Isolation of the 5′-flanking regions of vitellogenin promoter was performed by genome walking as previously described [24]. Sequence analyses were performed using BLAST (http://www.ncbi.nlm.nih.gov/blast/) and protein sequence alignments conducted using Vector NTI Advance 10 (Invitrogen). Putative transcription factor binding sites were identified using the web-based Conreal server (http://conreal.niob.knaw.nl/).

### Plasmid construction and transgenic *Drosophila melanogaster*

For each vitellogenin gene, a pair of primers was designed to amplify the region 2–3 kb upstream from the start codon (Table 1). PCR reactions proceeded with incubation at 94°C for 2 min, followed by 35 cycles consisting of 94°C for 30 s, 58°C for 30 s and 72°C for 2 min.

Each amplified putative promoter fragment was cloned upstream of the *GFP* coding sequence in pGlow (Invitrogen). Each promoter, together with *GFP* and the *BGH* polyadenylation sequence from pGlow was PCR-amplified and digested using Asc I and Fse I (for *Vg1* promoters) or Fse I (for *Vg2* promoters) and inserted into the Fse I site in pBac[3XP3-DsRed] to generate four constructs which each had the general organization: pBac[3XP3-DsRed-CxVg-promoter-GFP-BGH]. Each construct was injected into *D. melanogaster* embryos (*w* ^1118^) with the helper plasmid phsp-pBac [31]. Transgenic *D. melanogaster* were identified by red eye fluorescence using a Leica MZ95 microscope with DsRed filter (exciter HQ545/30x; emitter HQ620/60m) and lines established.

### RT-PCR gel analysis of GFP transcripts in transgenic *D. melanogaster*

*Drosophila* fatbodies were dissected from adult female transgenic flies and total RNA extracted using the SV Total RNA Isolation system (Promega). 10–15 transgenic female adults (3 to 5 days old) for each sample were processed for RNA extraction. To remove genomic DNA contamination, the DNA-free™ kit (Ambion) was employed following the manufacturer's protocol. Two micrograms of total RNA was used as template for first strand cDNA synthesis using M-MLV Reverse Transcriptase (Promega). A pair of *GFP* specific primers (Table 1) was used for PCR. The PCR reaction was 2 min at 94°C, followed by 35 cycles consisting of 94°C for 30 s, 50°C for 30 s, and 72°C for 4 min 30 sec, and a final 72°C extension for 10 min. No-RT controls were included with every reaction.

### Quantitative RT-PCR assays

To quantify expression levels of *GFP* driven by *Cx. tarsalis* vitellogenin promoters in transgenic *D. melanogaster*, we conducted quantitative real-time PCR using *GFP*-specific and the *rp49*-specific (single-copy nuclear gene) primer sets (Table 1). Quantitative PCR (qPCR) was performed using the Rotor-Gene Q Real-Time PCR System (QIAGEN) using the Rotor-Gene SYBR Green PCR Kit (QIAGEN). qPCR for both *GFP* and *rp49* proceeded with incubation at 95°C for 5 min, followed by 40 PCR cycles consisting of 95°C denaturation for 5 s, 60°C annealing for 5 s and 72°C extension for 5 s and a final melting curve analysis of the PCR product to verify the specificity and identity, with ramp from 60°C to 95°C, rising by 1°C each step and 90 s for the pre-melt conditioning on first step, 5 s for each step afterwards. We tested 5 samples per treatment and each sample was replicated 3 times. Threshold cycle (Ct) values of the target gene *GFP* and *rp49* were calculated for each replicate. Expression folds of *GFP* between different constructs of vitellogenin promoters or lines were calculated using the comparative CT method of relative quantization [32]. No-RT controls were included with every reaction. Expression values between lines were statistically compared by Kruskal-Wallis test with the Conover-Inman method for multiple comparisons using StatsDirect software (statsdirect.com).
